# Draft Genome Sequence of *Mycobacterium chimaera* Type Strain Fl-0169

**DOI:** 10.1128/genomeA.01620-16

**Published:** 2017-02-23

**Authors:** Stacy Pfaller, Vasily Tokarev, Collin Kessler, Christopher McLimans, Vicente Gomez-Alvarez, Justin Wright, Dawn King, Regina Lamendella

**Affiliations:** a Office of Research and Development, National Exposure Research Laboratory, U.S. Environmental Protection Agency, Cincinnati, Ohio, USA; b Department of Biology, Juniata College, Huntingdon, Pennsylvania, USA; c Office of Research and Development, National Risk Management Research Laboratory, U.S. Environmental Protection Agency, Cincinnati, Ohio, USA

## Abstract

We report here the draft genome sequence of the type strain *Mycobacterium chimaera* Fl-0169, a member of the *Mycobacterium avium* complex (MAC). *M. chimaera* Fl-0169^T^ was isolated from a patient in Italy and is highly similar to strains of *M. chimaera* isolated in Ireland, although Fl-0169^T^ possesses unique virulence genes.

## GENOME ANNOUNCEMENT

The *Mycobacterium avium* complex (MAC) contains several clinically relevant closely related species. The MAC, including *M. avium*, *M. intracellulare*, and *M. chimaera*, contains the most frequently isolated mycobacteria from humans in the United States, surpassing isolations of *Mycobacterium tuberculosis* and other species (1, 2). While *M. avium* and *M. intracellulare* have been studied for decades, less is known about *M. chimaera*, which was recently described in 2004 (3). The type strain of *M. chimaera*, Fl-0169, was isolated in Italy from a 56-year-old female patient with bronchiectstasis (3). The strain produced discordant identification results based on phenotypic and genotypic comparisons to its close relatives in the MAC, and it was classified as a new species, for which the name *M. chimaera* was proposed (3). Evidence suggests that *M. avium*, *M. intracellulare*, and *M. chimaera* are differently virulent and require species-level identification for targeted treatment (4). Therefore, a comparative genomic analysis of MAC species is critically needed to identify diagnostic targets that reliably differentiate species of MAC. Prolonged outbreaks of *M. chimaera* from heater-cooler units used in open chest surgery also drive the need for better diagnostic tools (5, 6). With treatment costs for *Mycobacterium* infections estimated to be >$1.8 billion annually in the United States (7), correct species identification may result in improved treatment selection, lower costs, and improved patient outcomes.

*M. chimaera* strain Fl-0169^T^ was obtained from the Leibniz Institute DSMZ (Braunschweig, Germany) and genomic DNA extracted using the MoBio PowerSoil DNA extraction kit (Mo Bio Laboratories, Solana Beach, CA). Paired-end 125-bp libraries were prepared using the Nextera DNA library preparation kit (Illumina, Inc., San Diego, CA) and sequenced on the Illumina HiSeq 2500 (Illumina, Inc.). Quality-filtered reads were *de novo* assembled using SOAP*denovo*2 (8) and Velvet (9). Contigs from both assemblers were combined using CISA (10) and genomes annotated using the Joint Genome Institute’s (JGI) Integrated Microbial Genomics (IMG) (11), the U.S. Department of Energy Systems Knowledge Database (Kbase; http://www.kbase.us), and MG RAST (12) annotation pipelines. Upon submission to IMG (13), scaffolds were broken into contigs, and coding genes were identified with Prodigal (14) and assigned a locus tag. Compared to other *M. chimaera* strains (15), several genes are unique to strain Fl-0169^T^, including the putative virulence genes anthranilate phosphoribosyltransferase (EC 2.4.2.18), sporulation initiation inhibitor protein Soj, and a 3-oxoacyl-(acyl-carrier-protein) synthase, KASII (EC 2.3.1.41) (16).

The Insert Species into Genome Tree application within KBase was used to compare *M. chimaera* strain Fl-0169^T^ to other genomes and revealed that *M. chimaera* strain Fl-0169^T^ is most similar to *M. chimaera* strain MCIMRL6. The draft genome of strain Fl-0169^T^ consists of 304 contigs and an *N*_50_ of 33,938 bp, for a full assembly size of 6,308,407 bp. The average coverage per contig was 18.27×. The G+C content per assemblage of the CISA-assembled genomes was calculated using EMBOSS (17) and averaged 69.6%. Other features include the presence of 6,462 protein-coding genes (4,715 genes with and 1,747 genes without functional prediction), three rRNA genes, and 43 tRNA genes.

### Accession number(s).

This whole-genome shotgun project has been deposited at DDBJ/ENA/GenBank under the accession number MRBR00000000. The version described in this paper is version MRBR01000000.
